# Effect of Latent Heat by Phase Transformation on the Thermal Behavior of Steel Billet during Heating

**DOI:** 10.3390/ma16247598

**Published:** 2023-12-11

**Authors:** Joong-Ki Hwang

**Affiliations:** School of Mechatronics Engineering, Korea University of Technology & Education, Cheonan 31253, Republic of Korea; jkhwang@koreatech.ac.kr; Tel.: +82-041-560-1642

**Keywords:** heating, latent heat, phase transformation, billet, temperature deviation

## Abstract

The effect of latent heat via phase transformation on the thermal behavior of a billet was investigated during the heating process. The latent heat of the billet strongly affected the temperature distribution of the billet during heating, although the heating rate of the billet was not high during the process. The temperature profile of the center region of the steel billet with latent heat had a strong flat shape compared with the other regions, as the heat supply to the center region was limited during the heating process owing to the finite thermal conductivity and mass effect of the billet. The latent heat by phase transformation typically occurred in the middle stage of heating, and the latent heat increased the temperature deviation of the billet during heating owing to the delay in the temperature rise at the center region of the billet. During the phase transformation of carbon steels during heating, the gas temperature needs to be low to reduce the temperature deviation or thermal stress of the billet. Industrial hot rolling mills are required to consider the latent heat by phase transformation of the billet to properly design the heating pattern for the billet. The heating pattern in the reheating furnace should be varied with the materials to obtain a high heating quality for the billet.

## 1. Introduction

Reheating furnaces are widely used in steel rolling mills to increase the billet, bloom, or slab temperature prior to rolling processes [1], as a hot rolling process has several advantages over a cold rolling process [2], especially for mass production. The hot-rolled plate and sheet are fabricated using the slab, whereas the hot-rolled wire, rod, and bar are manufactured using a billet or bloom [3,4,5]. Therefore, the billet, bloom, and slab are called semi-finished products. In addition, bolts, bearings, springs, tire cords, and bridge cables are fabricated from wire rod steels [6], and wire rod steels are manufactured from billets via hot rolling. Before hot rolling, the discharging temperature from a reheating furnace ranges from 900 °C to 1300 °C. Discharging temperature refers to the billet temperature before exiting the reheating furnace for hot rolling.

The heating pattern of the billet can be designed by considering a discharged target temperature along with acceptable uniformity, high productivity, thermal efficiency, low NO_x_ and CO_2_ emissions, and the mechanical properties of the product after rolling. To satisfy these concerns, numerical analyses with optimization methodologies for the heating pattern and temperature measurement of the billet have been typically used in many industrial and academic fields. In order to understand and improve the heating pattern and thermal efficiency of the billet/slab, abundant studies have been conducted using numerical analysis and temperature measurement.

Generally, there are three approaches to the thermal analysis of a billet/slab during the heating process, as summarized in Table 1. The first approach is to solve the full Navier–Stokes, energy conservation, and species equations governing the hot fluid flow and combustion in the reheating furnace using commercial software such as FLUENT or STAR-CD [7,8,9,10,11,12,13,14]. Kim and Huh [7] predicted the slab temperature in a walking-beam type reheating furnace based on three-dimensional (3D) computational fluid dynamics (CFD) analysis for solving the convection and radiation heat transfer using FLUENT software. Han et al. [13] simulated the slab temperature in a bench scale reheating furnace using 3D in-house code involving the standard *k-ε* model for turbulence, eddy dissipation for combustion, and the finite volume method (FVM) for radiation. Tang et al. [11] predicted the temperature distributions inside the reheating furnace and slab using a 3D CFD model involving turbulent interacting flow and combustion. Prieler et al. [14] compared the transient heating characteristics of a steel billet under air-fired and oxygen enriched gas conditions in a walking hearth-type furnace based on 3D CFD analysis. Although such a 3D full CFD analysis can offer thermal characteristics with fluid flow and combustion over the entire 3D furnace domain, there are several difficulties in treating the numerous governing equations, complex furnace structures, and uncertainty in the models; therefore, long computational time and high costs are required. Accordingly, this method has certain limits to implementation in an industrial rolling mill owing to the imbalance between accuracy and efficiency.

The second approach is to focus on the transient heat conduction in the billet/slab and the analysis of the radiative heat transfer of the furnace gas [15,16,17,18,19,20,21]. This approach typically uses a zone method. Kim [15] predicted the temperature distribution in the slab and the radiative heat flux on the slab surface in the reheating furnace based on two-dimensional (2D) unsteady heat transfer model using FVM. Han et al. [16] reported the transient radiative heating characteristics of slabs in a walking-beam type reheating furnace using a non-gray weighted sum of gray gas model (WSGGM) with 3D FVM. Emadi et al. [18] reported the effects of the convective heat transfer coefficient and furnace wall emissivity on the temperature distribution of a billet in a walking hearth-type reheating furnace based on a 3D mathematical model with the zone method and WSGGM. Chen et al. [21] presented a novel method to obtain the comprehensive radiative and convective heat transfer coefficients of a billet in a reheating furnace by combining a black box test with a mathematical model. They reported that this method reduces the calculation time and is suitable for on-line control compared with the typical zonal method. Although the fluid flow in the furnace is not involved in this approach, the thermal behavior of the billet can be identified with sufficient accuracy for engineering perspectives, and the computational costs and time are less than those of the full CFD approach.

The third approach only concentrates on the transient heat conduction in the billet/slab. This model uses the radiation-type total heat transfer coefficient in each zone of the reheating furnace based on a temperature measurement of the billet [22,23,24,25,26]. Accordingly, a temperature measurement of the billet is strongly required to determine the total heat transfer coefficient for this model. Ji et al. [23] determined the total heat transfer coefficient of a walking beam-type regenerative reheating furnace based on a 3D mathematical model and industrial temperature measurement data, then analyzed the effects of operational parameters such as productivity, fuel consumption, and slab charging temperature on the total heat transfer coefficient. Jang and Kim [26] obtained the emission factors for a billet reheating furnace using a 2D finite element method heat transfer model and furnace instrumented billet temperature data. Luo and Yang [25] estimated the total heat transfer coefficient in a walking beam-type reheating furnace by solving the inverse heat conduction problem. The computational time of this approach is much less than that of the first and second approaches mentioned above, and the prediction accuracy remains high when the model is validated with the measured temperature relative to those of the full- and semi-CFD approaches. Therefore, many industrial hot rolling mills use these types of models to predict and control the billet/slab temperature in the reheating furnace. The disadvantage of this approach is that experiments for temperature measurements need to be periodically conducted to confirm or modify the model parameters. The strengths and weaknesses of the three approaches mentioned above are summarized in Table 1.

Based on the experience of the author, the heating behaviors of steel billets in reheating furnaces are very different depending on the phase transformation behavior of the steels during heating in spite of their similar heating patterns. Although it is a report on cooling, Edalatpour et al. [27] showed that the latent heat due to phase transformation of steel delays the cooling rate during water cooling of a hot-rolled strip in a run-out table, indicating that the presence or absence of phase transformation of a material during cooling and heating affects the thermal behavior of the material. Plain carbon steels and austenitic stainless steels (STSs) can exhibit different heating behaviors because austenitic STSs do not undergo phase transformation during heating and cooling [28,29,30]. Accordingly, the influence of the latent heat on the temperature distribution of the billet during the heating process needs to be revealed in order to properly design a heating pattern for each steel. For example, in plain carbon steels, the latent heat generated by the phase transformation during heating is an endothermic reaction. Without considering this reaction it is difficult to accurately predict the temperature profile of steels during heating.

Although there have been many studies on the thermal behaviors of the slab/billet in reheating furnaces based on thermal engineering, few studies have investigated the influences of the material properties on the thermal behaviors of the billet/slab in a reheating furnace. However, based on the experience of the author, although the latent heat by phase transformation of metals can slightly affect the overall heating rate of the billet during heating owing to the relatively slow heating rate, it can affect the temperature deviation of the billet with a region owing to the large size of the billet, slab, and bloom. Therefore, the influence of latent heat by phase transformation on the thermal behavior of a billet during the heating process was investigated in detail to understand the heating behaviors of a billet in a reheating furnace. In particular, the effect of latent heat on the temperature uniformity of the billet was analyzed in detail, as the main requirement for a discharged billet from industrial reheating furnaces is to obtain the target temperature with acceptable uniformity.

## 2. Experimental Procedures for Heating

A temperature measurement test of the billet was conducted to determine the boundary conditions, i.e., the radiation type total heat transfer coefficient during heating, and to evaluate the accuracy of the present temperature prediction model. A laboratory electric heating furnace was used in the present heating test. It had overall dimension of 3.0 m × 1.8 m × 2.8 m and was equipped with two electric heating devices located on the top and bottom of the furnace. The gas temperature in the heating furnace was controlled using two thermocouples installed in the middle of the furnace. The heating furnace was heated to 960 °C, then the cold billet was charged in the furnace using a roller conveyor. The laboratory heating furnace was designed to maintain 960 ± 10 °C at all times during the experiment.

**Figure 1:**
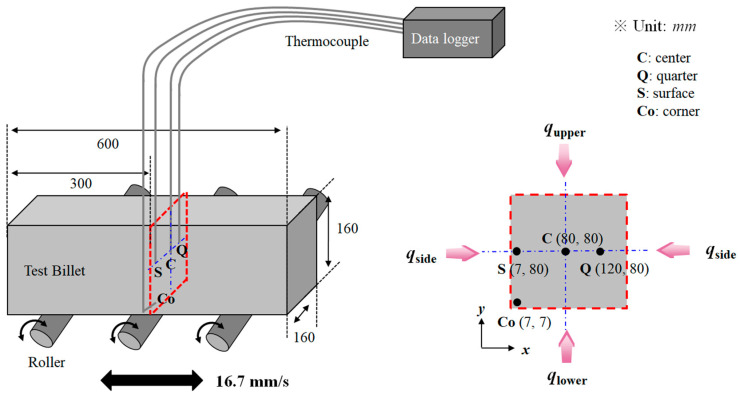
Schematic views of the physical domain for numerical simulation and temperature measurement points in the billet using four thermocouples.

An alloyed medium-carbon steel billet (AISI 4137) was adopted for the experiment because it is widely used in wire, rod, and bar products; its chemical composition is listed in Table 2. A single billet with a size of 160 mm × 160 mm × 600 mm was used, as shown in Figure 1. The initial temperature of billet was approximately 26 °C. The billet was supported by a conveyor roller in the heating furnace and periodically moved back and forth at a constant speed of 16.7 mm/s with a period of 107 s during heating. The effects of non-uniform heating along the longitudinal direction of the billet by the skid button and/or temperature deviation of gas in the reheating furnace [31,32] can be excluded in this experiment. Conveyor rollers were used for charging the billet into the furnace and discharging it from the furnace. In addition, to reduce the influence of oxide scale formation on the thermal behaviors of the billet [33], the billet was heated under nitrogen gas. The specific heating conditions are summarized in Table 3.

Figure 1 depicts the schematics of the measurement points of the temperature in the billet using thermocouples. Four regions of the billet were measured: the center (C), quarter (Q), surface (S), and corner (Co) regions. For instance, the thermocouple at the center region was mounted 80 mm under the upper surface of the billet and the thermocouple at the corner region was mounted 7 mm above the bottom surface. After drilling a 3.0 mm-diameter hole to a specific depth in the billet, a shielded K-type thermocouple with a 3.2 mm diameter was firmly embedded into the hole of the billet to reduce the thermal disturbances on the surface of the billet [34]. The outside of the thermocouple was slightly polished with sandpaper to ensure the solid attachment of thermocouples to the hole of the billet and a metal paste was used to eliminate the air gap in the hole of the billet [35]. A data recording apparatus gathered the measured temperatures with a sampling interval of 10 s. The data recorder apparatus was not mounted on the billet; instead, the temperature was measured through the long thermocouples outside the furnace, as shown in Figure 1. The ambient air temperature measured via a thermocouple was approximately 25 °C.

## 3. Numerical Model

### 3.1. Physical Problem and Materials

The heat transfer phenomenon of the billet was simulated using a 2D FVM. In the 160 mm × 160 mm square billet, the temperature difference along the longitudinal direction was much smaller than in the width or thickness direction of the billet. Therefore, the conductive heat transfer along the longitudinal direction of the billet during the heating process was ignored, as shown in Figure 1. However, it is impossible to ignore the heat transfer phenomenon along the width direction of the billet because of the cross-sectional shape of the square billet, indicating that 2D analysis is necessary for analyzing the temperature field of the billet.

**Figure 2:**
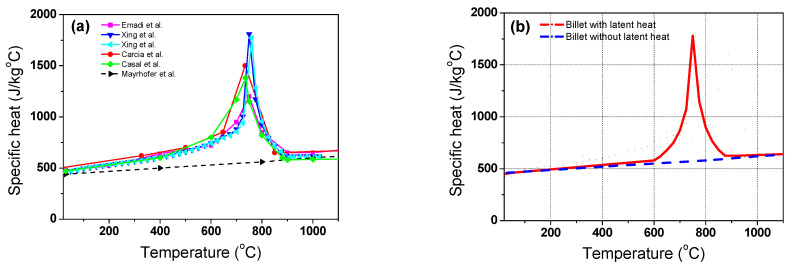
Comparison of (**a**) the specific heat of the steels from [18,29,36,37,38] and (**b**) the specific heat selected in this study.

The specific heat of the billet is dependent on the temperature. Figure 2a shows the specific heat of the steel as a function of temperature from several sources [18,29,36,37,38]. An abrupt change in the thermal properties appeared in the carbon steels due to the structure change of the steel from body-centered cubic (BCC) to face-centered cubic (FCC) structures [39,40]. It is known that the thermal properties of metals change with their chemical composition, grain size, and dislocation density [41]. In this study, the average specific heat of plain carbon steels with latent heat and STSs without latent heat was used, as shown in Figure 2b, because this study focused on the effect of latent heat on the temperature distribution of the billet during the heating process. Meanwhile, the density of the billet was assumed to be constant at 7850 kg/m^3^ [42,43,44] and the thermal conductivity of carbon steel was obtained from [18,29,36,37] as a function of temperature.

### 3.2. Governing Equations and Boundary Conditions

This study only concentrates on the transient heat conduction in the billet via the radiation-type heat transfer from the gas and wall in the reheating furnace. The third approach introduced in the introduction section was used [22,23,24,25]. The temperature field of the billet was governed by the transient conduction equation, as follows [45,46].

(1) $$\rho c_{p}(T)\frac{\partial T}{\partial t}=\frac{\partial }{\partial x}\left(k(T)\frac{\partial T}{\partial x}\right)+\frac{\partial }{\partial y}\left(k(T)\frac{\partial T}{\partial y}\right)$$

In the above, *ρ*, *c_p_*, *k*, and *T* are the density, specific heat, thermal conductivity, and temperature of the billet, respectively. In addition, *t* denotes the time and *x* and *y* are the coordinates in the 2D domain. Based on the experiment, the initial billet temperature was assumed to be uniform at 26 °C before it was charged into the furnace, as follows.

*T*(*x*, *y*, 0) = 26 °C (2)

The total heat flux (*q_t_*) on the surface of the billet was used as the boundary condition, as shown in Figure 1. During the heating process in the reheating furnace, the convective (*q_cv_*) and radiative (*q_r_*) heat fluxes needed to be considered, as follows [18,47]:

(3) $$q_{t}=q_{cv}+q_{r}$$

where *q_cv_* and *q_r_* are calculated using the following equations:

(4) $$q_{cv}=h_{cv}\left(T_{g}-T_{s}\right)$$

(5) $$q_{r}=\varepsilon \sigma \left(T_{g}^{4}-T_{s}^{4}\right)$$

where *h_cv_*, *ε*, and *σ* are the convective heat transfer coefficient, emissivity, and Stefan–Boltzmann constant, while *T_g_* and *T_s_* mean the gas temperature in the reheating furnace and the surface temperature of the billet, respectively. The 3D CFD analysis in the furnace is necessary to determine *h_cv_*; several researchers have used a constant value of *h_cv_* to evaluate the thermal behavior of the billet using the second and third approaches in introduction section [47,48]. However, it is known that radiative heat transfer is dominant during a high temperature reheating process; radiative heat transfer accounts for over 90% of the total heat transfer to the billet [13,15,18,45]. For example, Singh et al. [45] reported that the radiation heat transfer accounts for 94.28% of the total heat transfer in a walking beam-type reheating furnace. Emadi et al. [18] showed that approximately 90.3% of the heat flux to the billet depends on radiation. Therefore, the relatively small influence of convective heat transfer and several geometric factors for radiative heat transfer can be simply considered by using an emission factor (*φ*) for the radiation-type heat transfer, as follows.

(6) $$q_{t}=\varphi \sigma \left(T_{g}^{4}-T_{s}^{4}\right)$$

The *φ* used in this study is not a true emission factor or emissivity, instead being a simple tuning parameter depending on several heat transfer factors such as radiation from the gas and furnace wall, convection from the gas, and conduction from the conveyor rollers. Additionally, *φ* considers the influence of oxide scale formation on the billet surface, as such scale formation impedes the heat transfer from the furnace to the billet owing to the low thermal conductivity of the oxide scale [49]. Therefore, *φ* is generally called the total heat exchange factor [23,24,25]. The *φ* value can be determined by comparing the simulation billet temperatures with the measured billet temperatures. Physically, the constrain on *φ* is bounded between 0 and 1 [25] as follows.

0 ≤ *φ* ≤ 1 (7)

Accordingly, the total heat transfer coefficient (*h_t_*) in the reheating furnace was calculated as follows.

(8) $$q_{t}=h_{t}\left(T_{g}-T_{s}\right)$$

(9) $$h_{t}=\frac{\varphi \sigma \left(T_{g}^{4}-T_{s}^{4}\right)}{\left(T_{g}-T_{s}\right)}$$

The gas temperature around the billet in the reheating furnace was assumed to be constant owing to the small size of the reheating furnace and the lack of a skid beam and button under the billet used in this experiment. Therefore, the thermal radiation was assumed to be same on the four sides of the billet, that is, the upper, lower, right, and left sides of the billet, as shown in Figure 1.

### 3.3. Numerical Methods and Grid Independence

The transient heat conduction equation in Equation (1) was discretized in space (*x* and *y*) and time (*t*) using 2D in-house FVM code by incorporating a central difference and implicit schemes to simulate the heat conduction phenomenon of the billet, as well-described by Patnakar [50]. The discretized equations were solved iteratively using the tridiagonal matrix algorithm (TDMA) until the temperature contour within the billet satisfied a convergence criterion, as follows:

(10) $$max\left(\frac{\left|T_{i,j}-T_{i,j}^{old}\right|}{T_{i,j}}\right)\leq 10^{-6}$$

where $T_{i,j}$ and $T_{i,j}^{old}$ are the present and previous iteration values in the same time level, respectively.

**Figure 3:**
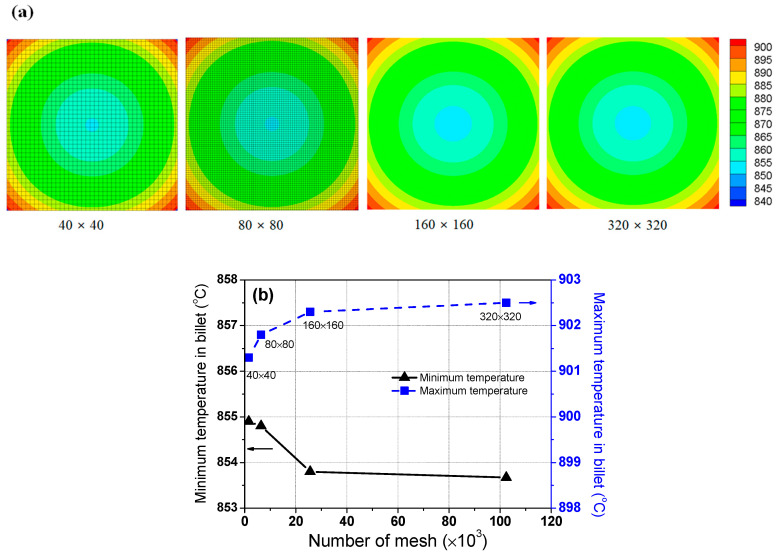
Comparison of (**a**) temperature contour (°C) and (**b**) maximum and minimum temperatures in the billet as a function of the element number.

A grid convergence test was performed using the four mesh systems: 40 × 40, 80 × 80, 160 × 160, and 320 × 320 elements. The temperature contours of the billet at the residence time of 50 min were compared for a time step of 30 s, constant *φ* of 0.5, and constant furnace gas temperature of 960 °C, as shown in Figure 3a. The post-processing for the temperature contour was performed with Tecplot (2014). It was found that the temperature contours in the billet of the four mesh systems were similar. In addition, the maximum and minimum temperatures in the billet were compared with the mesh system, as shown in Figure 3b. The minimum temperature decreased and the maximum temperature increased with increasing element number; however, the quantitative values were not significantly different with the element number, that is, the difference in the maximum temperatures in the billet with the element number was within 2 °C. The difference in the minimum temperatures with the element number showed similar results.

Based on the results of the temperature contours and the maximum/minimum temperatures in the billet, the four mesh systems showed similar results owing to the low heating rate of the billet. In particular, the mesh systems of 160 × 160 and 320 × 320 exhibited quite similar results. Therefore, the mesh system of 160 × 160 was adopted in this study. The mesh with 160 × 160 elements produced acceptable results compared to that with 320 × 320 elements at a lower computational time, leading to the use of this mesh system to simulate the temperature field of the billet during the heating process.

## 4. Results and Discussion

### 4.1. Model Parameter and Validation

**Figure 4:**
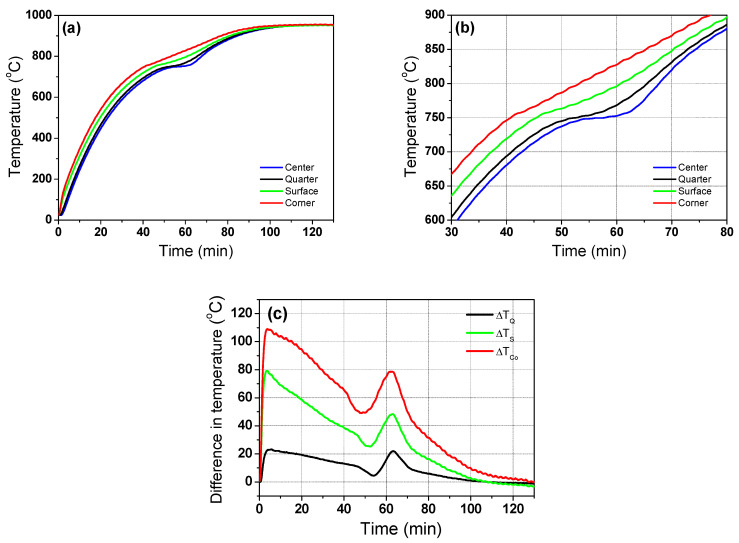
(**a**) Measured temperature profiles of the billet with the region, (**b**) enlarged section of the temperature profiles during the phase transformation, and (**c**) variation in measured temperature difference of the billet based on the center region with the residence time.

The numerical model was tuned by determining the optimum values of *φ* with the residence time of the billet. The value of *φ* can be determined by comparing the calculated billet temperatures with the measured billet temperatures as a function of the residence time. Figure 4a shows the measured temperature profiles at the four regions of the billet. As expected, the corner region exhibited the highest temperature and the center region exhibited the lowest temperature regardless of the residence time. The temperature of the quarter region showed a profile similar to that of the center region. The temperature in the surface region exhibited a moderate value between the center and corner regions. The temperature profiles had a lag point at approximately 750 °C owing to the latent heat by phase transformation of the carbon steel [40]. Figure 4b shows an enlarged section of the phase transformation. The center and quarter regions clearly exhibit flat temperature profiles at approximately 750 °C, whereas the corner and surface regions have a small lag in the temperature increase around 760 °C. The phase transformation occurs during the heating and cooling processes in plain carbon steels. During the heating process in plain carbon steels, ferrite, pearlite, bainite, and/or martensite are transformed into austenite. The latent heat is released during the phase transformation of steels [27,39] owing to the different specific heat of each phase (Figure 2b); therefore, the phase transformation can affect the thermal behavior of steels. For example, the heat is absorbed during heating; the amount of the heat absorption from the phase transformation is typically calculated using the enthalpy difference (Δ*H*) and volume fraction change (Δ*X*) of each phase in the defined Δ*t*, which is generally described as follows [51,52].

(11) $$q=∆H\frac{∆X}{∆t}$$

The Johnson-Mehl-Avrami type equation is typically used to calculate *X* during the cooling process [53]. The different behaviors of the temperature increase with the regions during the phase transformation, resulting in an increase in the temperature deviation in the billet, as shown in Figure 4c. In this figure, the temperature difference (Δ*T*) in each region was calculated by subtracting the center temperature (*T_C_*) from each point, such as quarter (*T_Q_*), surface (*T_S_*), and corner (*T_Co_*). For example, Δ*T* in the corner region was calculated as follows.

Δ*T_Co_* = *T_Co_* − *T_C_* (12)

The temperature difference increased abruptly at a residence time of approximately 60 min; the temperature difference between the billet corner and center was greater than 80 °C and that between the billet surface and center was approximately 50 °C. In addition, the temperature difference with the region in the billet was large in the initial stage of heating, as shown in Figure 4c. The temperatures at the corner and surface regions of the billet increased at a rapid rate as soon as the billet was charged into the reheating furnace, whereas the temperature at the center region rose relatively slowly in this initial heating stage.

**Figure 5:**
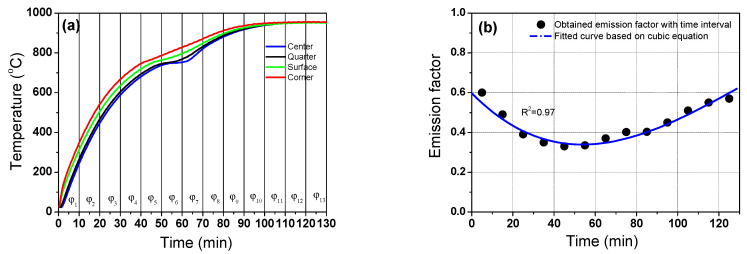
(**a**) Schematic description of the time interval for obtaining the emission factor and (**b**) variation in the obtained emission factor with the residence time used in this study.

The value of *φ* was determined by comparing the simulation temperatures with the measured billet temperatures at the corner region as a function of the residence time. To obtain the average *φ* for each residence time, the total residence time of 130 min was divided into thirteen time intervals of 10 min, as shown in Figure 5a, that is, the average *φ* was obtained every 10 min. Therefore, thirteen *φ* measurements was obtained with the residence time. It was assumed that *φ* was constant at the same residence time regardless of the location of the thermocouple. Under these assumptions, *φ* was obtained by minimizing the following objective function *S*(*φ*) in each of the thirteen time intervals, which was calculated as sum of the square error between the predicted temperatures (*T_p_*) with various *φ* and measurement temperatures (*T_m_*) [54]:

(13) $$S(\varphi )=\sum_{t_{i}}^{t_{f}}{\left(T_{p}\left(7,7,t;\varphi \right)-T_{m}\left(7,7,t\right)\right)}^{2}$$

where *t_i_* and *t_f_* refer to the initial and final time of the designed time interval; for example, *t_i_* and *t_f_* are 0 s and 10 s at the first time interval, and *φ*_1_ was determined using the equation above. In this way, *φ*13, the *φ* measurement at the last time interval of 120–130 s, was obtained sequentially. Figure 5b shows the obtained thirteen values of *φ* with the residence time. The derived *φ* values were fitted with a cubic equation (*φ*(*t*)), then *φ*(*t*) was inserted into the temperature prediction model based on Equation (6).

**Figure 6:**
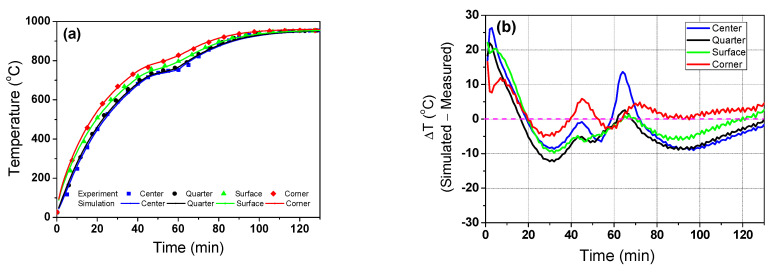
Comparison of (**a**) temperature profiles and (**b**) temperature differences between the experimental test and numerical simulation with the residence time.

Figure 6 compares the temperature profiles and temperature differences between the experimental test and numerical simulation with the residence time using the application of the optimum *φ*(*t*) in Figure 5b. As shown in Figure 1, the measured temperatures at the center, quarter, surface, and corner of the billet were compared with the simulated temperatures at the same positions. The predicted temperatures of the four regions exhibited reasonable agreement with the measured values, although the results of the numerical simulation were over-predicted in the initial stage and under-predicted in the final stage.

### 4.2. Influence of Latent Heat by Phase Transform

**Figure 7:**
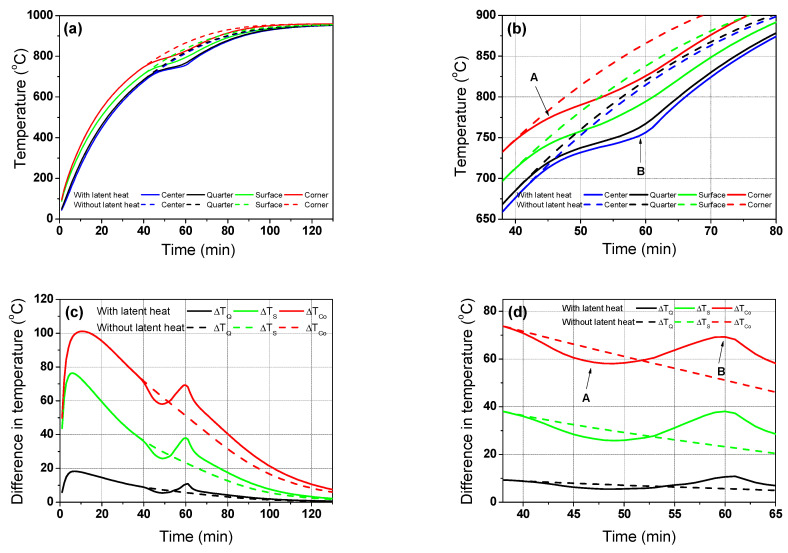
Comparison of (**a**,**b**) the temperature profiles and (**c**,**d**) the temperature difference based on the center region using the *w*LH and *wo*LH models with the residence time; (**b**,**d**) are the enlarged figures of (**a**,**c**), respectively.

**Figure 8:**
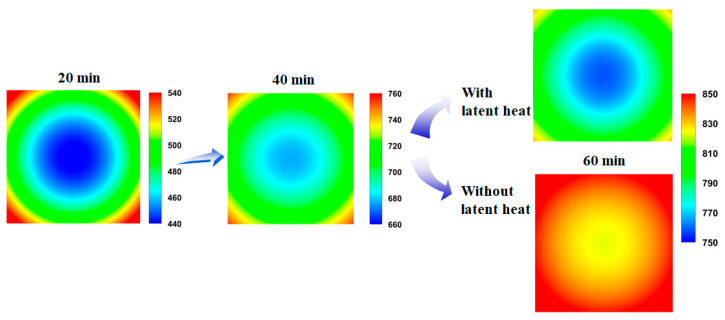
Comparison of the temperature contour (°C) of the billet using the *w*LH and *wo*LH models with the residence time.

To evaluate the influence of the latent heat by phase transformation on the temperature distribution of the billet during the heating process, the proposed temperature prediction model was executed for carbon steel with latent heat effects (*w*LH) and without latent heat effects (*wo*LH) based on Figure 2b. That is, while all other process and material conditions were kept constant, the two different *c_p_* values were inserted into the model and compared, as the peak in the *c*_p_ curve in Figure 2b is due to the enthalpy difference of the BCC and FCC structures caused by phase transformation [39,40]. Figure 7a,b compares the temperature profiles of the billet using the *w*LH and *wo*LH models. In the beginning, and prior to phase transformation, the two temperature profiles of the *w*LH and *wo*LH models are the same; however, the temperature profiles with the *w*LH model lie under the temperature profiles with the *wo*LH model owing to the heat absorption stemming from the crystal structure change from BCC to FCC, as clearly observed in the temperature contours shown in Figure 8. The latent heat by phase transformation typically occurs in the middle stage of heating.

Figure 7c,d shows a comparison of the temperature differences of the billet obtained using the *w*LH and *wo*LH models. The temperature difference of the *wo*LH model decreases with residence time, as the temperature difference between the furnace gas and billet surface decreases with increasing residence time. That is, the radiative heat flux from the furnace to the billet was reduced with the residence time because it is proportional to the fourth power of each temperature, as shown in Equation (5), indicating that the thermal behavior of the billet was very sensitive to the gas temperature in the reheating furnace. The temperature deviation with region of the billet mentioned above can be easily explained by the Biot number (*Bi*), which is defined by the ratio of the external heat transfer (*h_t_*) to the internal thermal conduction (*k*), as follows:

(14) $$Bi=\frac{h_{t}L}{k}$$

where *L* represents the mass effect of the material during heat transfer, for which the value in this study is 40 mm. The temperature deviation in the billet increased with an increasing *Bi* because the billet had insufficient time to transfer the external incoming heat to the inner region of the billet. Meanwhile, the temperature difference in the billet of the *w*LH model decreased prior to the start of the phase transformation, then increased abruptly as the phase transformation started, indicating that the phase transformation increased the temperature deviation of the billet during the heating process. The lower temperature difference in the billet of the *w*LH model relative to the *wo*LH model at point A in Figure 7d is highly related to the temperature lag of the corner and surface regions, as the temperatures of the corner and surface regions increases rapidly (Figure 7b), and the phase transformation of these regions started early relative to the center region, as shown in Figure 8. In addition, the higher temperature difference of the *w*LH model compared with the *wo*LH model at point B in Figure 7d is closely related to the temperature lag of the center region (Figure 7b). The temperature profile of the center region of the *w*LH model has a strong flat shape compared with the other regions, which is because the heat supply to the center region was limited during the heating process owing to the finite thermal conductivity and mass effect of the billet. Meanwhile, although the cross section of the billet is square, a circular temperature contour is shown in the billet as shown in Figure 8 because the temperature at the four corners rises quickly. In addition, because the heat fluxes on all four sides in the billet were assumed to be equal in this study, a symmetrical temperature contour was obtained. Asymmetric temperature contours will appear in the billet if different thermal boundary conditions are applied to the four surfaces of the billet. Strictly speaking, the heat flux on the four sides of a billet is not constant in a real furnace, therefore, a symmetrical temperature contour does not appear.

**Figure 9:**
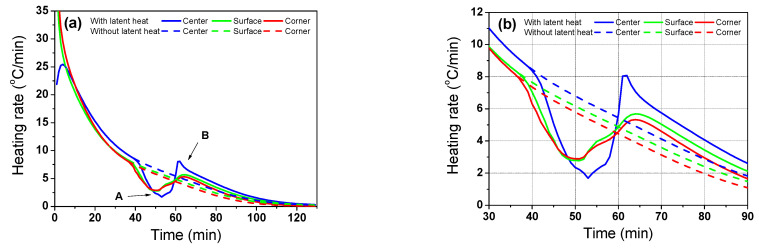
Comparison of the heating rate of the billet using the *w*LH and *wo*LH models with the region: (**a**) full and (**b**) phase transformation ranges.

Figure 9 compares the heating rates of the billet using the *w*LH and *wo*LH models with the region. The heat rate (*R*) was obtained using the measured time step (∆*t*), as follows.

(15) $$R=\frac{T_{i,j}^{t+∆t}-T_{i,j}^{t}}{∆t}$$

Overall, *R* exhibited a higher value in the initial stage of heating because the billet received strong thermal radiation from the gas and wall of the furnace originating from the high temperature difference between the billet and furnace environment, and then overall R decreased with the residence time. Meanwhile, each region of the billet exhibited different *R* values. In the initial stage of heating, the corner region had the highest *R* value, whereas the center region exhibited the lowest *R* value. Conversely, the corner region had a low *R* value, whereas the center region exhibited a high *R* value in the later stages of heating. As expected, the heating rate decreased with the residence time in the *wo*LH model, as the radiative heat transfer from the gas in the furnace to the billet decreases with residence time. However, the latent heat by phase transformation abruptly reduced the heating rate of the billet owing to the heat absorption of the billet during the phase transformation of the steels, particularly in the center region. After the phase transformation, the heating rate of the center region sharply increased, as the conductive heat transfer from the hot outer region to the inner region of the billet increased owing to the high temperature difference within the billet. In summary, the latent heat by phase transformation affects the heating rate of the billet based on the region, especially in the center region, resulting in temperature deviation of the billet. Therefore, industrial hot rolling mills are required to consider the latent heat caused by phase transformation of the billet in order to properly design the heating pattern for the billet. The heating pattern in the reheating furnace should be varied with the materials to obtain a high heating quality for the billet.

Meanwhile, a limitation of this study is that the microstructural analysis of the phase change was not evaluated quantitatively. The thermal behavior of the material can be different depending on microstructural changes due to phase transformation. Based on the experience of the author, an endothermic reaction occurred in this study as ferrite and pearlite structures changed to austenite during heating. Additional research is necessary to quantify the relationship between microstructural changes due to phase changes and endothermic reactions.

## 5. Conclusions

Based on a comprehensive study of the thermal behaviors of a billet during heating as a function of the latent heat by phase transformation, the following conclusions are drawn:

1. The latent heat by phase transformation strongly affected the temperature distribution of the billet during the heating process, although the heating rate of the billet was not high. The temperature profile of the center region of the steel with latent heat had a strong flat shape compared with the other regions, as the heat supply to the center region was limited during the heating process owing to the finite thermal conductivity and mass effect of the billet.
2. The latent heat by phase transformation typically occurred in the middle stage of heating, and the latent heat increased the temperature deviation of the billet during heating owing to the delay in the temperature rise at the center region of the billet.
3. Industrial hot rolling mills are required to consider the latent heat by phase transformation of the billet to properly design the heating pattern for the billet, that is, the heating pattern in the reheating furnace should be varied with the materials to obtain a high heating quality for the billet.

## Figures and Tables

**Table 1:** Comparison of three approaches to thermal analysis of a billet/slab during heating.

Approach	Strength	Weakness
3D CFD analysis in furnace including mass, momentum, energy, and species	Possible to analyse fluid flow in furnace Possible to secure optimal burner position and intensity of flame Possibility of convective heat transfer Possible to design a furnace before installation	Huge demands on computing resources Long calculation time Very expensive Requires commercial thermal analysis software Not suitable for on-line systems in industry
Temperature prediction based on analysis of billet and furnace gas/wall interaction	Low computation cost Short calculation time Sufficient accuracy for engineering perspectives	Unable to analyze flow and combustion in furnace Difficulties in dealing with convective heat transfer Requires significant understanding of radiative heat transfer mechanism
Temperature analysis of billet based on total heat transfer coefficient	Very low computation cost Very short calculation time Suitable for on-line systems in industry Easy to model except for boundary conditions	Need for furnace instrumented billet trial test of temperature Complicated determination of total heat transfer coefficient Requires different total heat transfer coefficient at each furnace Unable to analyze flow and combustion in furnace Impossible to design a furnace before installation

**Table 2:** Chemical composition of the alloyed medium-carbon steel used in the present heating experiment (wt.%).

C	Mn	Si	Cr	Mo	Fe
0.35	0.7	0.25	1.05	0.22	Balance

**Table 3:** Process conditions for the heating experiment in this study.

Condition	Parameter	Value
Billet	Material	Medium-carbon steel (AISI 4137)
	Dimension	160 mm × 160 mm × 600 mm
Furnace	Type	Laboratory electric heating furnace
	Overall dimension	3.0 m × 1.8 m × 2.8 m
	Gas condition	N_2_
	Billet support method	Conveyor rollers with oscillation
Heating conditions	Initial billet temperature	26 °C
	Gas temperature in furnace	960 °C
	Residence time	130 min
	Ambient air temperature	25 °C
Measurement of temperature	Number of measurement points	4 (center, quarter, surface, and corner)
	Type of thermocouple	K-type thermocouple with 3.2 mm-diameter
	Data recorder apparatus	GL 820 (Graphtec Corporation, Yokohama, Japan) with sampling interval of 10 s

## Data Availability

Data are contained within the article.

## Nomenclature

*Bi*	Biot number
*c_p_*	specific heat (J/kgK)
∆*H*	latent heat (kJ/kg)
*h*	heat transfer coefficient (W/m^2^K)
*k*	thermal conductivity of the billet (W/mK)
*L*	billet width (m)
*q*	heat flux (W/m^2^)
*R*	heating rate (K/s)
*S*	objective function
*T*	temperature (K)
*t*	time (s)
*x*, *y*	space coordinates (m)
Greek symbols	
*φ*	emission factor
*ρ*	density (kg/m^3^)
*σ*	Stefan–Boltzmann constant (5.56 × 10^−8^ (W/m^2^K^4^))
*ε*	emissivity
Subscript	
C	center
Co	corner
cv	convection
i	initial
f	final
g	gas in the reheating furnace
m	measured
p	predicted
Q	quarter
r	radiation
S	surface
t	total
